# Supplementary material for: Effect of proprioceptive neuromuscular facilitation (PNF) technique on posture, balance and gait characteristics of older adults with scapular dyskinesis: a randomized controlled trial
Source: PeerJ. 2025 Aug 14;13:e19718. doi: 10.7717/peerj.19718 (PMC12358108; doi:10.7717/peerj.19718)
Supplement: Supplemental Information 3 — Applied exercises protocol followed during treatment period for both groups (PNF+Ex, Ex). [file peerj-13-19718-s003.docx]

EXERCISE GROUP TREATMENT PROTOCOL (Ex)

| Exercise | Repetitions  (0-3 weeks) | Repetitions  (4-6 weeks) | Repetitions  (6-8 weeks) |
| --- | --- | --- | --- |
| Strengthening of abdominal muscles | 6 | 8 | 15 |
| Strengthening of back extensor muscles | 6 | 8 | 15 |
| Shoulder circles (backwards) | 6 | 8 | 15 |
| Scapular Retraction | 6 | 8 | 15 |
| Shoulder Shrugs | 6 | 8 | 15 |
| Chin Tucks | 6 | 8 | 15 |
| Posterior pelvic tilt | 6 | 8 | 15 |
| Pelvic Elevation (Bridge) | 6 | 8 | 15 |
| Standing Posture Alignment (with mirror) (5 mins) | 1 | 1 | 2 |
| Cat-Cow Stretch | 6 | 8 | 15 |
| TheraBand Row (Green-Medium Light) | 6 | 8 | 15 |
| Closed Stance Standing (30 sec) | 1 | 2 | 3 |
| Tandem Stance Standing (30 sec) | 1 | 2 | 3 |
| Uneven Surface Standing (30 sec) | 1 | 2 | 3 |
| Reciprocal Upper Limb Flexion Extension at Standing | 6 | 8 | 15 |
| Forward and Sideways Stepping | 6 | 8 | 15 |
| Weight Shifting Drills | 6 | 8 | 15 |
| Gait Training in Parallel Bars (2m walking) (lapses) | 2 | 2 | 3 |

Exercises were performed in pain limit.

PNF COMBINED WITH CONVENTIONAL EXERCISE GROUP TREATMENT PROTOCOL (PNF INTERVENTIONS ADDITION TO THE EXERCISES OF EXERCISE ALONE GROUP) (PNF+Ex)

| **PATTERN** | **TECHNIQUE** | Repetitions  (0-3 weeks) | Repetitions  (4-6 weeks) | Repetitions  (6-8 weeks) |
| --- | --- | --- | --- | --- |
| Anterior Elevation | Rhythmic Initiation | 6 | 8 | 12 |
|  | Repeated Contraction |  |  |  |
| Posterior Depression | Rhythmic Initiation |  |  |  |
|  | Repeated Contraction |  |  |  |
| Anterior Depression | Rhythmic Initiation |  |  |  |
|  | Repeated Contraction |  |  |  |
| Posterior Elevation | Rhythmic Initiation |  |  |  |
|  | Repeated Contraction |  |  |  |

Between exercises, 20 seconds resting periods were given to participants to prevent fatigue
